# Ecological significance of *Candidatus ARS69* and *Gemmatimonadota* in the Arctic glacier foreland ecosystems

**DOI:** 10.1007/s00253-023-12991-6

**Published:** 2024-01-15

**Authors:** Siddarthan Venkatachalam, Thajudeen Jabir, Puthiya Veettil Vipindas, Kottekkatu Padinchati Krishnan

**Affiliations:** Arctic Ecology and Biogeochemistry Division, National Centre for Polar and Ocean Research, Ministry of Earth Sciences (Govt. of India), Vasco-da-Gama, Goa India

**Keywords:** Genome-resolved metagenomics, Phylogenomics, *Candidatus ARS69*, *Gemmatimonadota*, Biogeochemical processes

## Abstract

**Abstract:**

The *Gemmatimonadota* phylum has been widely detected in diverse natural environments, yet their specific ecological roles in many habitats remain poorly investigated. Similarly, the *Candidatus ARS69* phylum has been identified only in a few habitats, and literature on their metabolic functions is relatively scarce. In the present study, we investigated the ecological significance of phyla *Ca. ARS69* and *Gemmatimonadota* in the Arctic glacier foreland (GF) ecosystems through genome-resolved metagenomics. We have reconstructed the first high-quality metagenome-assembled genome (MAG) belonging to *Ca. ARS69* and 12 other MAGs belonging to phylum *Gemmatimonadota* from the three different Arctic GF samples. We further elucidated these two groups phylogenetic lineage and their metabolic function through phylogenomic and pangenomic analysis. The analysis showed that all the reconstructed MAGs potentially belonged to novel species. The MAGs belonged to *Ca. ARS69* consist about 8296 gene clusters, of which only about 8% of single-copy core genes (*n* = 980) were shared among them. The study also revealed the potential ecological role of *Ca. ARS69* is associated with carbon fixation, denitrification, sulfite oxidation, and reduction biochemical processes in the GF ecosystems. Similarly, the study demonstrates the widespread distribution of different classes of *Gemmatimonadota* across wide ranges of ecosystems and their metabolic functions, including in the polar region.

**Key points:**

• *Glacier foreland ecosystems act as a natural laboratory to study microbial community structure.*

• *We have reconstructed 13 metagenome-assembled genomes from the soil samples.*

• *All the reconstructed MAGs belonged to novel species with different metabolic processes.*

• *Ca. ARS69 and Gemmatimonadota MAGs were found to participate in carbon fixation and denitrification processes.*

**Supplementary Information:**

The online version contains supplementary material available at 10.1007/s00253-023-12991-6.

## Introduction 

The Arctic glacier foreland (GF) ecosystems are formed due to glaciers retreat; recent studies show that they continuously expand in the polar and alpine regions (Ficetola et al. 2021; Li et al. 2019a). When a glacier retreats, new landscapes are exposed, locked under the ice for several years (Venkatachalam et al. 2021). Microorganisms initially colonize these new terrains and play a pivotal role in nutrient recycling and ecosystem functioning by participating in biogeochemical processes (Mapelli et al. 2018; Sun et al. 2023; Venkatachalam et al. 2021). In the present study, we focused on microbial phyla, *Ca. ARS69* and *Gemmatimonadota*, to decipher their potential ecological role in the Arctic GF ecosystems.

Current progress in genome sequencing technologies and the development of novel bioinformatics tools revolutionized microbial ecology research by circumventing the necessity to isolate and characterize microbial groups from complex environmental samples (Parks et al. 2017). The genome-based phylogenetic taxonomic framework has been used to describe several novel microbial lineages, to elucidate key microbially mediated metabolic processes even among complex ecosystems (Nayfach et al. 2021). The Genome Taxonomy Database Toolkit (GTDB-Tk) has reclassified several microbial candidate phyla due to their distinct lineages (Chaumeil et al. 2022). One such group is *Ca. ARS69*, a monophyletic group from the phylum *Gemmatimonadota*. To date, the *Ca. ARS69* phylum does not have any culturable representative. As of June 2023, only one MAG, reconstructed from the samples collected in the Indian Ocean (Sunagawa et al. 2015), was publicly available in the NCBI database. However, the MAG was only 55.49% complete, thus making it challenging to elucidate several critical metabolic processes associated with this phylum. Recently, Pallen et al. (2022) proposed a new taxonomic name *Ca. Tufoliota* for *Ca. ARS69*. However, the International Committee on Systematics of Prokaryotes (ICSP) has yet to approve the proposed name.

The *Ca. ARS69* closest lineage, phylum *Gemmatimonadota*, was considered as cosmopolitan group accounting for about 0.2–6.5% of total diversity and identified as one of the top eight abundant bacterial phyla in the soil (Venkatachalam et al. 2021). To date, only six culturable representatives, namely, *Gemmatimonas aurantiaca, G. phototrophica*, *G. groenlandica*, *Roseisolibacter agri*, *Gemmatirosa kalamazoonesis*, and *Longimicrobium terrae*, have been isolated from this particular phylum (Aldeguer-Riquelme et al. 2022). Species *L. terrae* are associated with the class of *Longimicrobia*, whereas all the remaining isolates belong to the class of *Gemmatimonadetes*. Previous reports have shown that *G. aurantiaca* can reduce N_2_O, whereas *G. phototrophica* is known to participate in anoxygenic phototrophic mechanisms due to the presence of photosynthesis gene cluster (PGC) in their genome (Park et al. 2017). These PGC gene clusters are very similar to the one present in the phylum *Proteobacteria*, and it is suggested that the phototrophic mechanism in the *Gemmatimonadota* originated from *Proteobacteria* through horizontal gene transfer mechanisms (Mujakić et al. 2021). Recent studies have also shown the pivotal role of *Gemmatimonadota* in oxidizing greenhouse gases like N_2_O, hydrogen, and participating in the nitrogen and sulfur cycling process (Mujakić et al. 2021; Mujakić et al. 2022). The 16S rRNA gene–based metabarcoding approach revealed its global presence across several ecosystems, including marine, terrestrial, glacier, deep sea, hydrothermal vents, and wastewater (Aldeguer-Riquelme et al. 2022). A recent study from Aldeguer-Riquelme et al. (2022) deciphered the distribution, abundance, and ecological functions of *Gemmatimonadota* class *PAUC43f*, which are known to be prevalently distributed across marine ecosystems. However, the ecological role of *Gemmatimonadota* groups especially in GF ecosystems is relatively scarce. Similarly, there is no literature on the genetic diversity and metabolic functions of *Ca. ARS69* phylum, which led us to investigate in detail about the genetic, metabolic diversity and ecological functions of these two phyla in the present study.

## Materials and methods

### Sample collection and data description

In this study, three surface soil samples were collected across the Midtre Lovénbreen GF during the summer season of 2019 (Venkatachalam et al. 2021). The genomic DNA was extracted from these three soil samples using PureLink™ microbiome DNA purification Kit (Invitrogen, USA). Three metagenomic shot-gun libraries from the extracted DNA were prepared using the NEBNext® Ultra™ DNA Library Prep Kit (New England Biolabs). The quality of the libraries was assessed using Tapestation (Agilent Technologies) and sequenced using paired-end sequencing (2 × 150 bp) chemistry on an Illumina HiSeq X10 platform. In the present study, we have also used publicly available metagenomic datasets (https://www.ebi.ac.uk/ena/browser/view/PRJEB41174) from three different GF ecosystems (Varliero et al. 2021; Nash et al. 2018), namely, Midtre Lovénbreen glacier (Svalbard), Russell glacier (Greenland), and Storglaciären (Sweden). The MAGs belonging to *Gemmatimonadota* were reconstructed from these reference datasets. The metadata of all the samples used in the present study is listed in Supplementary table S1. The sequence datasets generated in this study are publicly available under the following NCBI Bio project ID: PRJNA944391.

### Metagenome assembly and reconstruction of MAGs

The sequence datasets were first subjected to a quality check using the FastQC program, followed by the removal of low quality and trimming of adapter sequences using “iu-filter-quality-minoche,” implemented in Illumina-utils v2.11 package in Anvio v7.1 (Eren et al. 2021; Eren et al. 2013). Similarly, any sequences matching the human genome were removed from the dataset using Bowtie2 v2.3.5 (Langmead and Salzberg 2012). The sequence reads were assembled into longer contiguous sequences by a *de novo* assembly approach using metaSPAdes v3.15.2 (Nurk et al. 2017). The assemblies were further quality-checked using MetaQUAST v5.0.2 (Mikheenko et al. 2015), followed by automated binning was performed using three separate binning tools, namely, CONCOCT v1.1.0 (Alneberg et al. 2013), maxbin2 v2.2.7 (Wu et al. 2015), and METABAT2 v2.12.1 (Kang et al. 2019) to generate MAGs in the Anvio v7.1 pipeline. The MAGs were manually refined by the anvi-refine program using anvi’o interactive interface by exploiting differential coverage, tetranucleotide frequency, and marker gene content information to remove contamination/redundancy. Then, DAS_Tool v1.1.2 was employed to sort the MAGs which contain completeness of > 50% and redundancy of < 10% among the three binning MAG sets. The MAG abundance was calculated by considering the mean coverage of each contig divided by that sample’s overall mean coverage using an anvi-profile program (Eren et al. 2021). Furthermore, a non-metric multidimensional scaling (NMDS) plot and ANOSIM analysis were carried out on the MAG abundance data to study the distribution patterns using PRIMER v7 (Clarke and Gorley 2015).

### Taxonomic classification, phylogenomic, pangenomic, and metabolic functional analysis of MAGs

All the reconstructed MAGs were subjected to average nucleotide identity (ANI)–based taxonomic classification using GTDB-Tk tool v1.5.1 against reference database V207 (Chaumeil et al. 2019, 2022). If any MAGs that do not classify ANI cut-off of 95% were considered as novel species. Furthermore, to facilitate the comparative phylogenomic analysis of *Ca. ARS69* and *Gemmatimonadota*, we have downloaded publicly available MAG datasets belonging to *Gemmatimonadota* (*n* = 1278) and *Ca. ARS69* (*n* = 1) phyla using the NCBI genome portal. The downloaded MAGs were again reclassified GTDB-Tk tool v1.5.1 as described above. Similarly, the quality of the genomes was also screened by checkM tool v1.2.2 (Parks et al. 2015) (Supplementary table S2). The classification revealed a further 3 MAGs belonged to Ca. ARS69 phylum. However, these MAGs were only about 55 to 77% complete (Supplementary table S2). Despite their low completeness, we have included those MAGs in the analysis as they are the only available source belonging to *Ca. ARS69*. While among *Gemmatimonadota*, only those MAGs with high completeness (> 85%) and less redundancy (< 10%) were subjected to dereplication using dRep v3.0.0 tool at 95% ANI to select only representative MAGs at the species level. The resulting reference MAGs (*n* = 240) belonged to diverse ecosystems like freshwater (*n* = 73), terrestrial (*n* = 71), marine (*n* = 64), wastewater (*n* = 17), deep sea (*n* = 10), groundwater (*n* = 7), glacier (*n* = 6), alkaline salt lake (*n* = 4), and wood decay (*n* = 1). The reference MAGs, along with reconstructed MAGs from GF ecosystems (*n* = 13), were further subjected to phylogenomic analysis using PhyloPhlAn v.3.0 package (Asnicar et al. 2020) with the following parameters (“-diversity high,” “-d phylophlan,” “--accurate”). The generated phylogenetic tree was visualized using Anvio v7.1. Based on the phylogenomic analysis, only those MAGs and isolated genomes closely affiliated with reconstructed MAGs were further subjected to pangenomic analysis. The study was carried out as per the pangenomics workflow (https://merenlab.org/2016/11/08/pangenomics-v2/) using Anvio v7.1. The metabolic capacity of the MAGs was analyzed by the METABOLIC v4.0 program to identify the potential metabolic genes corresponding to carbon, nitrogen, sulfur cycles using 143 custom HMM profiles against the curated reference databases of KEGG, TIGRfam, and Pfam (Zhou et al. 2022). We also analyzed the potential community-level functions and relative abundance of these MAGs in the glacier foreland ecosystems.

## Results

### Metagenome assembly and genomic characteristics of the reconstructed MAGs

The paired-end metagenomic reads were assembled by the *de novo* approach for the samples collected across Midtre Lovénbreen GF along with publicly available metagenomic datasets from similar Arctic GF ecosystems (Varliero et al. 2021), namely, Russell glacier (Greenland) and Storglaciären (Sweden). The metagenome assembly resulted in an average of 6437 Mb length for each sample with approximately 70,303 contigs with > 2.5-kb length (Supplementary table S3). A list of metagenome assembly characteristics for each sample, including L50, N50, and the number of genes annotated, was given in Supplementary table S3. We have used both automated and manual binning refinement processes (visually inspecting contigs through an interactive interface) along with dereplication strategies to reconstruct high-quality MAGs belonging to *Gemmatimonadota* (*n* = 12) and *Ca. ARS69* (*n* = 1) from the metagenome assemblies through genome genome-resolved metagenomic approach. The MAGs completeness ranged from 86 to 99%, with the redundancy ratio between 0 and 9%, whereas size ranged from 2.3 to 5.1 Mb (Supplementary table S4). Similarly, the GC content of the MAGs was from 60.9 to 68%. All the reconstructed MAGs (*n* = 13) were of medium to high-quality draft, with most of them containing all the ribosomal rRNA genes (5S, 16S, 23S rRNA, and tRNAs) within their genome according to MIMAG genomic standards (Bowers et al. 2017). The MAG abundance was also found to be varied, with few MAGs being exclusively present only in a few of the samples (Supplementary fig. S1, Supplementary table S4). For example, ML2_Bin_1 and ML1_Bin_2 were highly abundant only in Midtre Lovénbreen GF, while G1_Bin_29 and G5_Bin_00004 were prevalently distributed across Russell and Storglaciären GFs. The NMDS analysis also showed that MAG abundance patterns significantly differed across three GF ecosystems (*P* < 0.002, *R* = 0.561; Fig. S1B).

### Phylogenomic analysis of MAGs

**Fig. 1:**
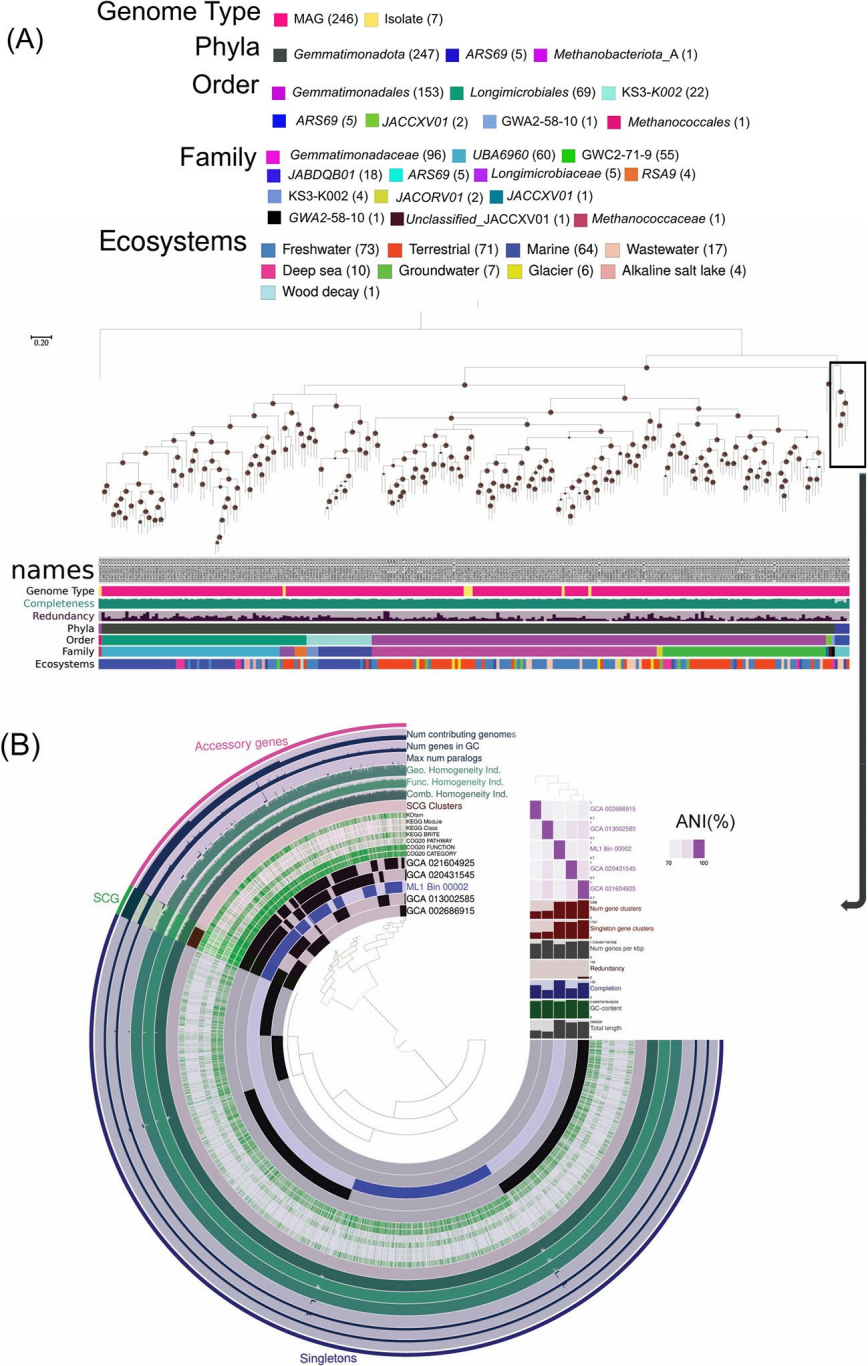
**A** Phylogenomic analysis of *Ca. ARS69* and *Gemmatimonadota* based on 253 MAGs and isolate genomes (publicly available) recovered among diverse ecosystems. The bar graphs represent the genome type, completeness, redundancy. Isolate genome *Methanococcus maripaludis* (GCF_002945325) was used as an outgroup. The color codes represent different taxonomic lineages and ecosystems of the recovered MAGs. Bootstrap values are represented in the nodes of the tree. The taxonomic names in the supplementary table S2 were arranged according to the names in the phylogenomic tree. **B** Pangenomic analysis of *Ca. ARS69* MAGs (*n* = 5). The circle diagram was based on presence/absence of the 8296 gene clusters (GCs) where it is represented by each layer for a single genome. Black and blue bars indicate presence of GCs, whereas grey indicates absence. The GCs are categorized based on their frequency among all the genomes as single copy core genes, accessory genes, and singletons. The other outer rings represent the presence of different KEGG, COG functions among each genome, where ANI values are also represented as heatmaps

The phylogenomic analysis was carried out by using the PhyloPhlAn v.3.0 package (Asnicar et al. 2020), which placed all the MAGs belonging to *Ca. ARS69* phyla as a distinct lineage outside of *Gemmatimonadota* cluster (Fig. 1A). The reconstructed MAG (ML1_Bin_00002) belonging to *Ca. ARS69* clustered within the family of *ARS69* MAGs which was recovered from the marine sediment (GCA_013002585) and wastewater (GCA_021604925) ecosystems (Fig. 1A; Supplementary table S2). The genome-based taxonomic classification based on ANI showed that this MAG was classified up to only family level, thus potentially belonging to novel genera (Supplementary table S4). Similarly, the MAGs belonging to *Gemmatimonadota* were grouped into four major clusters (Fig. 1A). Most of them belonged to the order of *Longimicrobiales* (aka SG8-23) and KS3-K002, which was mainly recovered from marine, freshwater, and deep-sea ecosystems. While MAGs belonging to the order of *Gemmatimonadales* were widespread across terrestrial (*n* = 61), deep sea (*n* = 4), freshwater (*n* = 57), wastewater (*n* = 15), glacier (*n* = 6), groundwater (*n* = 6), marine ecosystems (*n* = 4) (Supplementary table S2). The reconstructed MAGs from GF ecosystems, ML2_Bin_1, SW3_Bin_2 and G5_Bin_3, were phylogenetically closely associated with the family of GWC2-71-9, which is often found in terrestrial, freshwater, marine and deep-sea ecosystems (Zheng et al. 2022). The MAGs, SW3_Bin_16, SW5_Bin_8, SW6_Bin_27, ML3_Bin_9, SV1_Bin_3, were clustered with genus UBA4720 which have been previously identified from terrestrial and freshwater ecosystems (Supplementary table S4). Similarly, SW4_Bin_66 clustered with genus JACDCY01; G1_Bin_29 clustered with genus AG11; G5_Bin_4 clustered with genus FEN-1250. The ecological significance of these identified genera has not been studied previously.

## Pangenomic analysis of MAGs

**Fig. 2:**
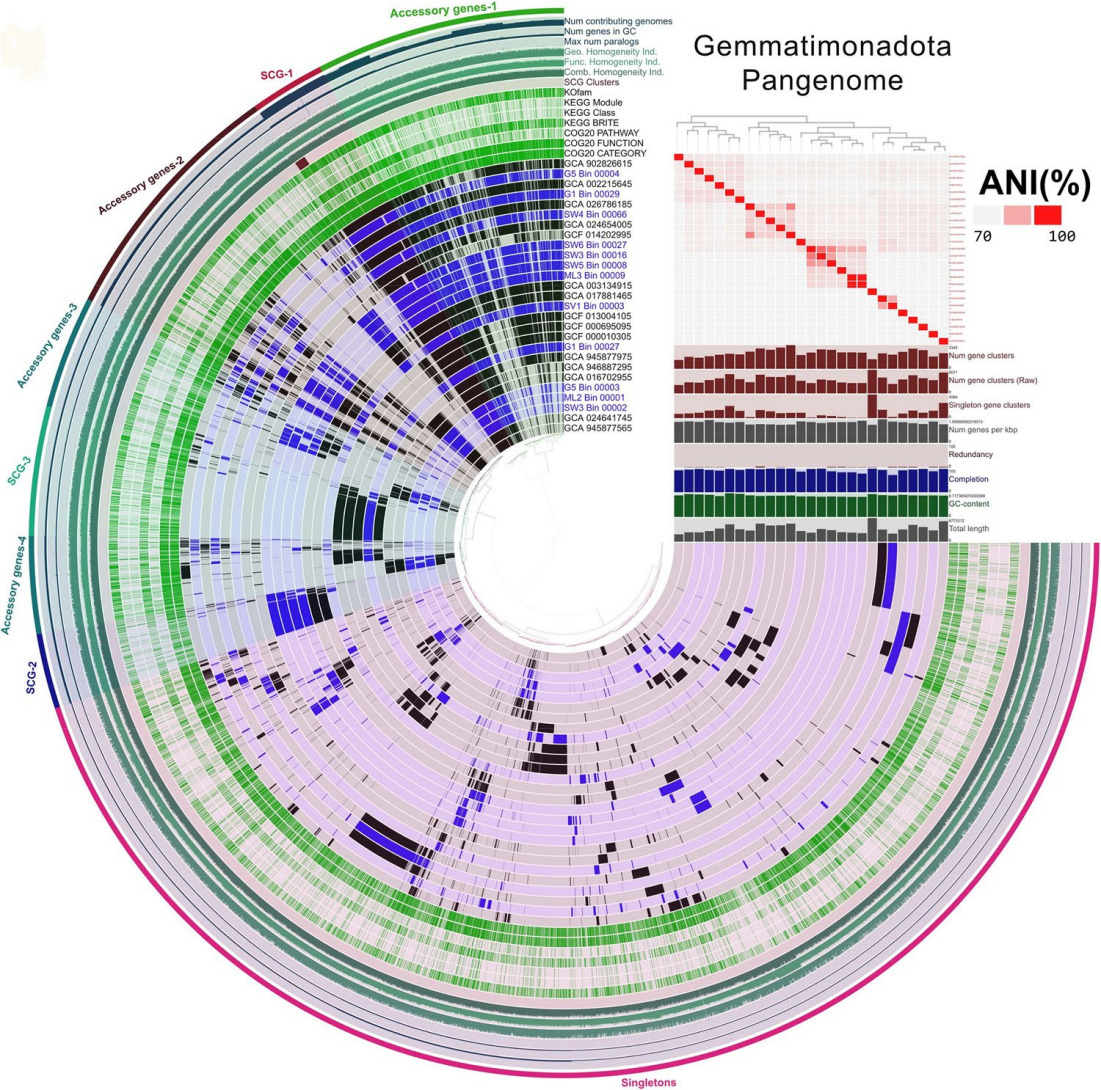
Pangenomic analysis of *Gemmatimonadota* MAGs and isolate genomes (*n* = 27). The circle diagram was based on the presence/absence of gene clusters (GCs), represented by each layer for a single genome. Black and blue bars indicate the presence of GCs, whereas grey indicates absence. The MAGs reconstructed from the present study were highlighted using the blue font. The GC is categorized based on frequency among all the genomes as single copy core genes, accessory genes, and singletons. The other outer rings represent the presence of different KEGG and COG functions among each genome, where ANI values were also portrayed as heat maps

The pangenomic analysis was conducted using ANIVIO v.7.1 software (Eren et al. 2021). The investigation revealed that all the MAGs (*n* = 5) belonging to phylum *Ca. ARS69* consist of 8,296 gene clusters (12,925 genes), of which only about 8% of single-copy core genes (*n* = 980) were shared among them (Fig. 1B and Supplementary table S5). This observation suggests either the presence of incomplete reference genomes within this group or the existence of a distinct lineage that has yet to be discovered. The analysis further revealed the distribution of accessory genes (i.e., genes present in more than one genome but not in all the genomes), which consist of 43% (*n* = 5571) of the total pangenome, whereas 49% of the genes (*n* = 6368) were categorized as singletons (i.e., genes only present in a single genome) among the studied genomes (Supplementary table S5). Altogether, with the distribution of diverse metabolic gene clusters along with average nucleotide identity (ANI), it is evident that the MAGs among this phylum are more divergent (ANI varied from 69 – 73%) and potentially belonging to novel microbial lineage (ANI < 85 % cut off; Supplementary table S6). For the *Gemmatimonadota* pangenome analysis, only those MAGs and isolate genomes that are found to be phylogenetically closely affiliated with reconstructed MAGs belonging to the phyla of *Gemmatimonadota* were used (Fig. 2). The study showed *Gemmatimonadota* (*n* = 27) was formed of 4 primary groups with about 10,424 gene clusters (63,837 genes). These four distinct groups within *Gemmatimonadota* comprised about 22% of single-copy core genes and 48% of accessory genes (Supplementary table S7). The ANI values among the studied genomes varied from 69.7% to 99% (Supplementary table S8). Based on KEGG, most of the metabolic functions among these two bacterial phyla, *Ca. ARS69* and *Gemmatimonadota*, were carried out mainly by core and accessory gene clusters (Supplementary table S5 & S7).

## Metabolic functions of the reconstructed MAGs

**Fig. 3:**
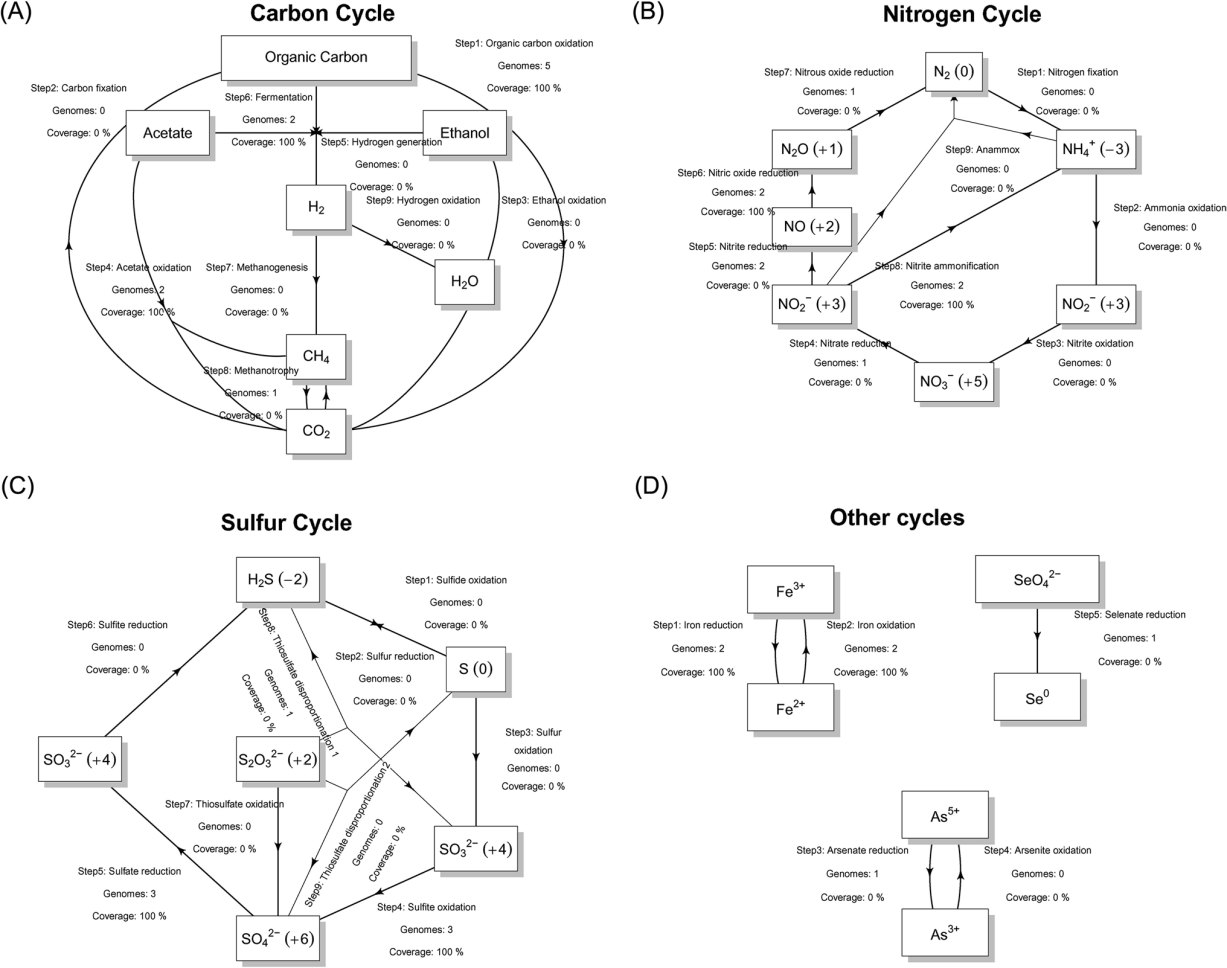
Potential ecological role of *Ca. ARS69* in biogeochemical processes. The metabolic pathways associated with **A** the carbon cycle, **B** the nitrogen cycle, **C** the sulfur cycle, and **D** other cycles were represented in the schematic diagram. The number of MAGs containing each metabolic pathway and their coverage (%) was provided for each carbon, nitrogen, sulfur, and other cycles

**Fig. 4:**
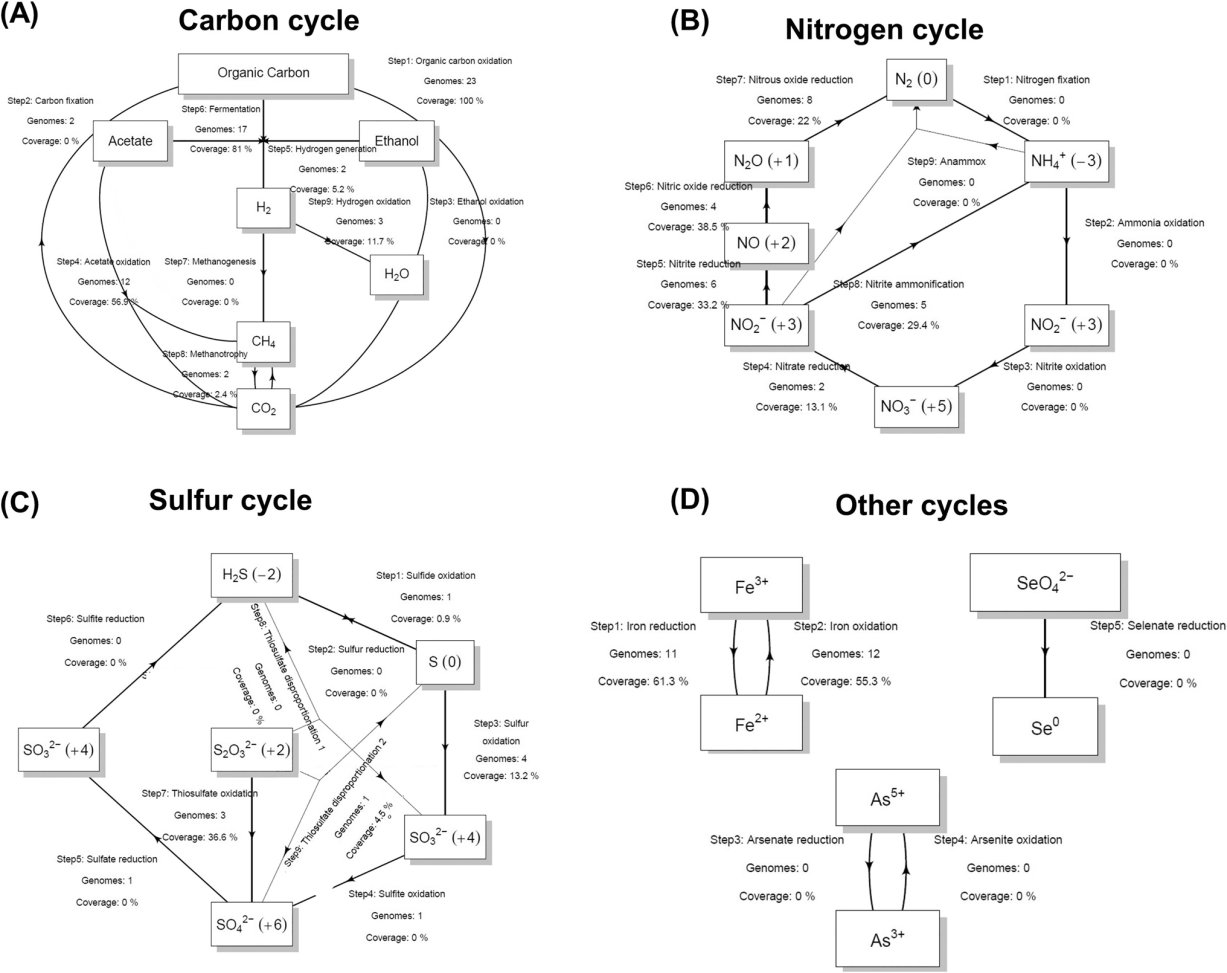
Potential ecological role of MAGs belonging to *Gemmatimonadota* in the biogeochemical processes. The metabolic pathways associated with **A** the carbon cycle, **B** the nitrogen cycle, **C** the sulfur cycle, and **D** other cycles were represented in the schematic diagram. The number of MAGs containing each metabolic pathway and their coverage (%) was provided for each carbon, nitrogen, sulfur, and other cycles

Among the carbon cycling pathways, the metabolic genes encoding organic carbon oxidation were highly abundant (coverage 100%) across all the *Ca. ARS69* MAGs. The metabolic potential for acetate oxidation (coverage 100%), fermentation (coverage 100%) and methanotrophy were also found to be prevalent in the *Ca. ARS69* (Fig. 3). Among the nitrogen cycles, metabolic genes encoding for complete denitrification processes (nitrate, nitrite, nitric oxide, nitrous oxide reduction) and nitrite ammonification (anammox) genes, where in the case of sulfur cycles, genes encoding for sulfite oxidation, sulfate reduction and thiosulfate disproportionation were identified in the *Ca. ARS69* (Fig. 3). Biologically, H_2_S is produced via sulfite reduction, thiosulfate disproportionation, and sulfur reduction, which were all identified exclusively in the MAG, ML1_Bin_2. A few of the MAGs within this group were also found to contain additional metabolic potential for iron oxidation and reduction processes (ML1_Bin_2, CA_021604925), arsenate reduction (CA_021604925) and selenate reduction processes (CA_021604925). Among the analyzed *Gemmatimonadota* MAGs (*n* = 27), many of them were found to contain genes encoding for organic carbon oxidation (*n* = 23, coverage = 100%), fermentation (*n* = 17, coverage = 81%), acetate oxidation (*n* = 12, coverage = 56.9%), and methanotrophy (*n* = 2, coverage = 2.4%) (Fig. 4). The metabolic genes encoding carbon fixation, hydrogen generation, and hydrogen oxidation were also found exclusively in a few MAGs belonging to this group which is not identified within the *Ca. ARS69* phylum. Similarly, among the sulfur cycles, genes encoding sulfide and sulfur oxidation were exclusively found within MAGs belonging to *Gemmatimonadota* (Fig. 4). Where in the case of nitrogen cycles, all of the metabolic processes that are identified within the *Ca. ARS69* phylum were also present among the MAGs of *Gemmatimonadota* (Fig. 4). Similarly, iron oxidation (*n* = 11, coverage 61.3%) and reduction process (*n* = 12, coverage 55.3%) were also prevalently identified within the MAGs belonging to *Gemmatimonadota*, while none of them contain metabolic genes associated with arsenate and selenate metabolism. From the above analysis, it is evident that these MAGs belonging to *Gemmatimonadota* could be able to catabolize a wide range of organic and inorganic sources available in the GF ecosystems.

## Discussion

The Arctic GF ecosystems are known to chronologically consist of different soil types at various developmental stages and inhabit distinct microbial community structures mediating key biogeochemical processes (Mapelli et al. 2018). Due to rapid global warming, these GF ecosystems are rapidly evolving, which influences the succession of microbial community and vegetation structure across GF ecosystems (Kim et al. 2017; Schütte et al. 2009; Varliero et al. 2021; Venkatachalam et al. 2021, 2024). However, the rate of these successional dynamics can vary among different GF ecosystems, which is influenced by many variables such as climatic conditions, topography, bedrock composition, and nutrient availability (Mapelli et al. 2018). Microbial communities and their associated biogeochemical processes might be specific to each GF (Varliero et al. 2021). The physicochemical factors, total nitrogen, and total organic carbon mainly shaped the microbial community structure across Russell GF (Greenland), whereas chronological distance from glacier ice edge and soil pH shaped the community structure in the Midtre Lovénbreen and Storglaciären GFs (Varliero et al. 2021; Venkatachalam et al. 2021; Nash et al. 2018). These differences may be due to the rate at which these GFs retreat in the current global warming scenario. Though several studies reported diversity and succession of microbial communities across the different GF ecosystems (Kim et al. 2017; Schuette et al. 2010; Schütte et al. 2009; Venkatachalam et al. 2021), only a few studies investigated functions and processes associated with microbial communities (Nash et al. 2018; Varliero et al. 2021). In particular, reconstructing MAGs directly from soil samples provides unique opportunities to study individual community members, their metabolisms, and their survival strategies (Maggiori et al. 2021). In particular, the samples collected from the extreme environmental region are often much more difficult to cultivate in the laboratory in comparison to other tropical regions. In the present study, we specifically focused on reconstructed MAGs belonging to two microbial phyla, namely, *Ca. ARS69* and *Gemmatimonadota*, to decipher their potential ecological functions.

One of the main notable findings from our study was the recovery of the first high-quality MAG belonging to *Ca. ARS69* from the recently deglaciated region of Midtre Lovénbreen GF, Svalbard. The phylogenomic analysis of all the available MAGs within this group, along with its closets monophyletic group *Gemmatimonadota*, confirmed its distinct lineage (Fig. 1A). The genomic diversity among this group was also found to be highly diverse based on the ANI similarity and shared only few gene clusters between the analyzed MAGs in the study (Fig. 1B). The analysis showed several metabolic genes associated with organic carbon oxidation, acetate oxidation, fermentation, methanotrophy, denitrification, nitrate ammonification, sulfite oxidation, sulfate reduction, iron oxidation, iron reduction, and arsenate reduction processes among the *Ca. ARS69* group (Fig. 3). The GF ecosystems are generally known to be oligotrophic (Dong et al. 2022; Venkatachalam et al. 2021), while the presence of diverse metabolic processes within this group suggests the prevalence of autotrophic mechanisms for their survival (Wong et al. 2020). Previous studies have also identified many novel microbial groups from such oligotrophic environments with potential ecological functions. For example, novel MAGs belonging to *Ca. Nitrosopolaris* and *Ca. Eremiobacterota* were reported from the polar and alpine ecosystems and found to have genes associated with carbon and nitrogen fixation processes (Ji et al. 2021; Pessi et al. 2022; Venkatachalam et al. 2024). Similarly, metabolic genes related to nitrogen fixation, and rock weathering processes have been investigated across four different Arctic GF ecosystems (Nash et al. 2018; Schuette et al. 2010; Varliero et al. 2021). Most microbial taxa identified in the recently deglaciated environment are generally inhabited by pioneering microbial groups that act as a seed bank for further soil developmental processes (Rime et al. 2016; Sun et al. 2023). The identification of *Ca. ARS69* phyla with diverse metabolic capabilities in the recently deglaciated environment suggests its crucial role in shaping the biogeochemical processes in GF ecosystem.

The phylogenomic and pangenomic analysis of *Gemmatimonadota* showed the existence of four different groups within their phyla. Interestingly, on contrary to previous reports (Aldeguer-Riquelme et al. 2022; Mujakić et al. 2021), the present study showed evidence that marine and deep ocean lineage is also closely affiliated with the terrestrial subgroup of *Gemmatimonadota* (Figs. 1A and 2). The reconstructed MAGs from GF ecosystems, ML2_Bin_1, SW3_Bin_2, and G5_Bin_3, were phylogenetically closely associated with the family of GWC2-71-9, which is often found in terrestrial, freshwater, marine, and deep-sea ecosystems (Zheng et al. 2022). All the reconstructed MAGs from this study belonged to the class of *Gemmatimonadetes* and potentially novel taxa (Supplementary table S4). Bacterial taxa belonging to this class are known to be prevalent in different types of soil environments and wastewater treatment plants (Mujakić et al. 2021; Mujakić et al. 2022). In the Arctic GF ecosystems, taxa belonging to *Gemmatimonadota* were found to be composed of up to 6.5% of total bacterial diversity (Venkatachalam et al. 2021). Previous studies based on 16S rRNA gene-based amplicon sequencing also showed the prevalent distribution of this group in the polar cold, dry desert soils (Cary et al. 2010; Chan et al. 2013). Despite numerous existing literatures on the diversity and distribution profiles of *Gemmatimonadota*, still little is known about their metabolic strategies to delineate their role in the environment. The present study showed evidence of potential metabolic processes associated with *Gemmatimonadota* bacterial groups in the Arctic GF ecosystems through genome-resolved metagenomics. One of the notable findings was the presence of metabolic genes encoding for hydrogen generation and hydrogen oxidation processes in the MAGs (Fig. 4), which delineates the involvement of *Gemmatimonadota* bacterial groups in scavenging atmospheric hydrogen for their chemosynthetic metabolic processes. Previous studies have shown this chemosynthesis process was prevalently identified in the bacterial taxa belonging to *Verrucomicrobiota*, *Eremiobacterota*, *Proteobacteria*, *Chloroflexota*, and *Bacteroidota* (Ortiz et al. 2021; Ray et al. 2022; Ray et al. 2020). The genes encoding sulfide and sulfur oxidation processes were also identified only in the *Gemmatimonadota* MAGs, which were absent in the *Ca. ARS69*. Similar abundant bacterial groups associated with sulfur metabolic processes were also previously identified in polar soils (Li et al. 2019b; Ortiz et al. 2021; Xue et al. 2020). However, both of these groups were found to contain several genes encoding for nitrogen cycling, iron oxidation, and iron reduction processes. Previous studies have also shown iron-driven denitrification processes in many autotrophic microorganisms (Li et al. 2023; Mapelli et al. 2011). Several previous studies have also reported the prevalence of microbial taxa associated with iron oxidation, iron reduction, and denitrification processes in the recently deglaciated regions of GF ecosystems (Nash et al. 2018; Varliero et al. 2021). From the study, it is evident that both of these groups will play crucial roles in shaping the Arctic GF ecosystem functioning.

In the present study, we reconstructed the first high-quality draft genome belonging to *Ca. ARS69* phylum, which could participate in diverse metabolic processes in the Arctic GF ecosystems. We also deciphered the phylogenetic diversity of Gemmatimonadota and their widespread distribution across several ecosystems, including its potential role in shaping the GF ecosystems. Our results expand the diversity of these two phyla and their putative role in carbon, nitrogen, and sulfur biogeochemical cycles. The reconstructed MAGs presented in this study will also serve as a valuable resource for future investigations into the dynamics, ecophysiology, and evolutionary processes within the Arctic GF ecosystems.

## Supplementary information

ESM 1 (PDF 364 kb)

ESM 2 (XLSX 17302 kb)

## Data Availability

The present study did not generate codes, and mentioned tools used for the data analysis were applied with default parameters unless specified otherwise.

## Data availability

The sequence datasets generated in this study are publicly available under the following NCBI Bio project ID: PRJNA944391. The reconstructed metagenome-assembled genomes were publicly available and can be downloaded using the following link: https://figshare.com/articles/dataset/Ecological_significance_of_Candidatus_ARS69_and_Gemmatimonadota_in_the_Arctic_glacier_foreland_ecosystems/23599014
