# Supplementary material for: Ecological significance of Candidatus ARS69 and Gemmatimonadota in the Arctic glacier foreland ecosystems
Source: Appl Microbiol Biotechnol. 2024 Jan 15;108:128. doi: 10.1007/s00253-023-12991-6 (PMC10789658; doi:10.1007/s00253-023-12991-6)
Supplement: Supplementary file 1 [file 253_2023_12991_MOESM1_ESM.pdf]

**Journal name:** Applied Microbiology and Biotechnology Journal

**Manuscript Title :** Ecological significance of ***Candidatus ARS69*** and *Gemmatimonadota* in the Arctic glacier foreland ecosystems

**The name(s) of the author(s) :** Siddarthan Venkatachalam*, Thajudeen Jabir, Puthiya Veettil Vipindas, Kottekkatu Padinchati Krishnan

**The affiliations:** Arctic Ecology and Biogeochemistry Division, National Centre for Polar and Ocean Research, Ministry of Earth Sciences (Govt. of India), Vasco-da-Gama, Goa, India.

**The e-mail address :** venkatachalam@ncpor.res.in **(Corresponding author)**

**Phone:** +91-08322525626

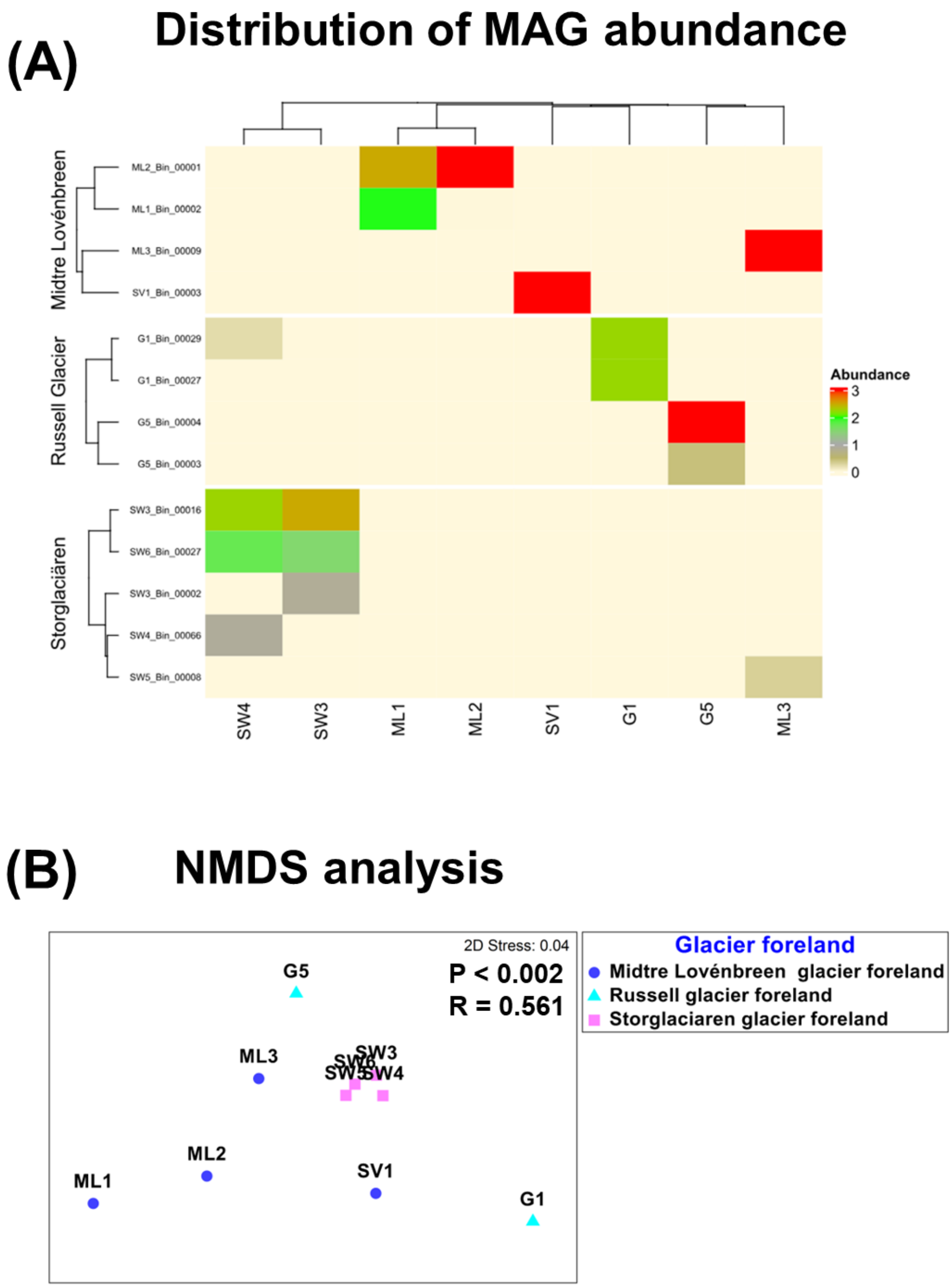


**Supplementary Fig. S1.** (A) Heatmap showing the abundance of reconstructed among the sampling sites. The rows represent the MAGs grouped by the hierarchical clustering method. Similarly, in the column, the samples were grouped by hierarchical clustering. The MAG abundance was normalized to the Z score and presented as “Abundance”. The heatmap was generated using the R package “complexheatmap” in R version 4.1.3. (B). NMDS analysis showing the MAG abundance distribution patterns among three GF ecosystems.
